# Acceptability of a Prime Vendor System in Public Healthcare Facilities in Tanzania

**DOI:** 10.34172/ijhpm.2020.90

**Published:** 2020-06-14

**Authors:** August Kuwawenaruwa, Fabrizio Tediosi, Emmy Metta, Brigit Obrist, Karin Wiedenmayer, Vicky-Sidney Msamba, Kaspar Wyss

**Affiliations:** ^1^Ifakara Health Institute, Dar es Salaam, Tanzania.; ^2^Swiss Tropical and Public Health Institute (Swiss TPH), Basel, Switzerland.; ^3^University of Basel, Basel, Switzerland.; ^4^School of Public Health and Social Sciences (SPHSS), The Muhimbili University of Health and Allied Sciences (MUHAS), Dar es Salaam, Tanzania.; ^5^Health Promotion and System Strengthening (HPSS) Project, Dodoma, Tanzania.

**Keywords:** Acceptability, Supply Chain, Prime Vendor, Tanzania

## Abstract

**Background:** Pharmaceutical supply chain management in low- and middle-income countries has received substantial attention to address the shortage of medicines at peripheral facilities. The focus has been on health system interventions, including the establishment of public-private partnerships (PPPs). In 2014, the United Republic of Tanzania began implementing the Jazia prime vendor system (Jazia PVS) with a contracted private wholesale supplier to complement the national medicines supply chain in public facilities. Few studies have investigated the acceptability of such a prime vendor system. This study analyses factors that contributed to the acceptability of Jazia PVS introduced in Tanzania. We used qualitative analytical methods to study experiences of Jazia PVS implementers in 4 districts in mid-2018.

**Methods:** Data were drawn from 14 focus group discussions (FGDs), 7 group discussions (GDs) and 30 in-depth interviews (IDIs) with a range of actors involved in Jazia PVS. The study analysed 7 acceptability dimensions as defined in the acceptability framework by Sekhon et al. Framework analysis was adopted to summarise the results using a deductive and an inductive approach.

**Results:** The findings show that participants’ acceptability of Jazia PVS was influenced by the increased availability of essential medicines at the facilities, higher order fulfilment rates, and timely delivery of the consignment. Furthermore, acceptability was also influenced by the good reputation of the prime vendor, close collaboration with district managers, and participants’ understanding that the prime vendor was meant to complement the existing supply chain. Intervention coherence, experienced opportunity cost and intervention burden, affective attitude and self-efficacy were also important in explaining the acceptability of the Jazia PVS.

**Conclusion:** In conclusion, the most critical factor contributing to the acceptability of the Jazia PVS was the perceived effectiveness of the system in achieving its intended purpose. Districts purchasing directly from the prime vendor have a policy based on the possibility to increase availability of essential medicines at peripheral facilities in a low income setting; however, it is crucial to select a reputable and competent vendor, as well as to abide by the contractual agreements.

## Introduction

The pharmaceutical supply chain in low- and middle-income countries faces multiple challenges, such as the existence of falsified medicines, underfunding, affordability, weak transparency, weak mechanisms of accountability, and inefficiencies in medical prescriptions to the patients.^1-3^ Consequently, pharmaceutical supply chains have received substantial attention,^4^ focusing on health system interventions such as those for redesigning and optimising the public sector pharmaceutical supply chain,^4-7^ including increasing financial resources allocated to the supply chain, introduction of incentives for staff to act effectively,^8-10^ staff training to improve pharmaceutical supply chain management skills,^11,12^ improvement of the supply chain process and procurement procedure such as centralising/decentralising purchasing of drugs, improvement of health information management systems to monitor and inform purchases, as well as infrastructure improvement and communication.^13-15^ Another policy introduced in several countries is the establishment of public-private partnerships (PPPs)^5,13^ to complement existing medicines supply chain systems.^4,16,17^ PPPs have been reported to increase the availability of essential medicines in peripheral areas in low-income settings^18-21^ to improve order fulfilment rates, to control drug costs, and to increase satisfaction among programme users.^20^

The successful deployment of new devices, interventions, or PPPs within the health system usually requires high acceptability by both implementers and beneficiaries of the intervention.^15,22-24^ The degree of acceptance by beneficiaries affects not only the successful implementation of the PPP, but also the effectiveness of the intervention.^25^ In any intervention, low acceptability implies that the intervention may not be delivered as intended, thus impeding the overall effectiveness of the intervention.^26^ Several studies have explored the acceptability of health system interventions.^23,24,27^ However, few studies have been conducted to assess the acceptability of interventions in the health supply chain.^15,28^ For example, Shieshia et al that, found after the adoption of technology within the pharmaceutical supply chain in Malawi, there was a reduction of time spent in attaining medicines from collection points, and there was improved drug availability and accountability.^15^ Furthermore, individual affective attitude and intervention coherence in terms of ease of use, safety and reliability contribute to the acceptability of an intervention.^28,29^

In Tanzania, the Medical Store Department (MSD) is the main supplier of medical commodities (drugs, medical equipment, medical supplies) to all public healthcare facilities and some of the private non-profit facilities. MSD receives funds from the Ministry of Finance to supply the facilities with the medical commodities based on facility quantification, and then MSD delivers the consignment directly to the respective facility. In recent years, MSD has been facing difficulties which hinder its efficiency in supplying all facilities, including delays in accessing funds from the Ministry of Finance, inaccurate forecasting of drug needs at facility levels, thefts, as well as ineffective systems for fulfilling back-ordered items.^30^ Districts and healthcare facilities have complementary funds that are earmarked for purchasing medical commodities from private pharmaceutical suppliers (retailers or wholesalers) when MSD is out-of-stock.^31^ Still, the purchase of complementary medical commodities has been reported to be poorly managed.^31^ In 2012, a survey was conducted to assess the availability of medicines in all the public healthcare facilities in Dodoma region, where it was found that order fulfilment rate from the MSD was about 60%, with facilities experiencing an average stock-out rate of 40% in essential medicines.^32^

In 2014, the United Republic of Tanzania started implementing a PPP programme in 3 pilot regions (Dodoma, Morogoro, and Shinyanga) in which all public healthcare facility orders for missing healthcare commodities at the MSD are pooled at the district level, and then purchased from one contracted supplier, the prime vendor. The system was named ‘ *Jazia prime vendor system* – *Jazia PVS*’ as it complements in situations where the MSD has failed to supply needed healthcare commodities. There is only one prime vendor for each region, contracted for 2 years. Jazia PVS is anchored in the structures of the regional health administration and it is overseen, supported and managed by mandated administrative structures such as the regional administrative secretary, the regional prime vendor coordinating office, regional health management teams, and council health management teams (CHMTs). The concept and set-up of the Jazia PVS was funded by the Swiss Agency for Development and Cooperation (SDC) through the Health Promotion and Systems Strengthening (HPSS) project in the period from January 2014 to July 2019. HPSS project staff oversee the operations of the Jazia PVS together with government officers. Selection of the prime vendor followed a tender process where all private retailers and wholesalers in Tanzania were invited to bid. They were evaluated and assessed, based on various criteria such as prices of commodities, financial capacity, staff, vehicles, quality assurance, systems in place for the procurement, and storage facility. After evaluation, the successful vendor was contracted to supply commodities for a given region. Orders for medical commodities from public hospitals, health centres and dispensaries are consolidated and purchased at previously agreed prices from the preferred and contracted private wholesale supplier, the prime vendor. After accounting for missing items from MSD each quarter, facility in-charges quantify the remaining needs and share with the district pharmacist who consolidates a list of needs across facilities and submits them to the vendor. In turn, the vendor delivers the consignment at the district headquarters where facilities are responsible for picking-up their consignment. Framework contracts with prime vendors at the regional level are established for 2 years. The contractual agreement specifies that the vendor deliver consignments at the district headquarters within 14 working days from confirmed date of receipt of an order from the respective districts. Emergency order(s) should be delivered within 5 working days. In addition, the contracts specify that the prime vendor be paid within 22 days by the respective healthcare facility. Medical commodities purchased from the prime vendor are financed with funds from national insurance schemes (Community Health Fund and National Health Insurance Fund), out-of-pocket payments, and basket funds.^18^ Jazia PVS pools financial resources available to districts to address shortages of essential medicines at public facilities. Continuous monitoring in the Jazia PVS pilot regions showed that this complementary prime vendor system has been effective in increasing the availability of essential medicines in public health facilities.^18^ Consequently, in 2018 the government decided to roll out the Jazia PVS to all 26 regions of Tanzania’s mainland.

This study analyses factors that contributed to the acceptability of the Jazia PVS introduced in Tanzania. It is part of a broader assessment of Jazia PVS that include other studies: one study analysed how accountability mechanisms contributed to the performance of Jazia PVS in Tanzania,^33^ and another study looked at the cost and cost-drivers of setting up Jazia PVS in public healthcare facilities in Tanzania (A. Kuwawenaruwa, K. Wyss, K. Wiedenmayer K, F. Tediosi, unpublished data, 2020).

## Key Messages

**Implications for policy makers**

- Establish and maintain a routine monitoring system, for example, through the district health information system which allows tracking effects and results of innovation such as Jazia prime vendor system (Jazia PVS) on the pharmaceutical supply.
- Establish and communicate a transparent and well understandable framework guiding supply chain improvements to relevant stakeholders, namely district authorities, health workers, and the private sector.
- Emphasise the confidence in and compliance to the rules and procedures guiding the public-private partnerships (PPPs) by district pharmacists, healthcare providers, district and regional managers.
- Strengthen coordination between the entities responsible for managing quantification, forecasting, procurement, and distribution of the health commodities at the regional, district, and facility levels to ensure timely availability and proper use of health commodities at the facilities.

**Implications for public**

Public-private partnerships (PPPs) in supply chains have the potential to improve service delivery; however, it is crucial to select a reputable and competent vendor, as well as abide by the contractual agreement.

## Methods

###  Conceptual Framework 

The concept of acceptability has several definitions.^23,27,34^ In general, it refers to the degree to which the intended programme beneficiaries, as well as those involved in implementing a given intervention, consider it to be congruent with cultural beliefs, and values.^23,24,35^ In this study we adopt a definition of acceptability as defined by Sekhon et al, * ‘a multi-faceted construct that reflects the extent to which individuals, as well as institutions affected by an intervention directly or indirectly, consider it to be suitable, based on their expectations, or experienced reasoning and emotional reactions to such intervention.’*^23^ Sekhon et al gave specific dimensions to be considered when analysing the acceptability of interventions in the context of health systems.^22,23^ Table highlights the 7 dimensions of acceptability and their definitions; (*i*) perceived effectiveness, (*ii*) affective attitude, (*iii*) intervention coherence, (*iv*) ethicality, (*v*) self-efficacy, (*vi*) opportunity costs, and (*vii*) experienced intervention burden.^23^ The ‘ *perceived effectiveness*’ corresponds to the degree to which an intervention successfully reaches the desired beneficiaries and produces the expected or desired output. ‘ *Affective attitude*’ refers to the emotional reaction or feeling of an individual towards the object of the attitude.^36^ An individual makes a judgment about, or has feelings toward, the Jazia PVS before or after taking part in it; such a judgment can be positive or negative (like or dislike of the attitudinal object). *‘Intervention coherence’* denotes a personal belief that the intervention is logical, consistent, and makes sense. ‘ *Intervention ethicality*’ represents the notion that the intervention is in accordance with the rules/values concerning the right practices or conduct, particularly the standards of a profession. ‘ *Self-efficacy*’ has been defined by Bandura as an individual’s self-belief in his/her capacity to execute behaviours necessary to produce specific performance attainments.^37^ Individual confidence and self-assessment influence the level of effort that is executed in achieving the goals, together with the likelihood of achieving particular level of behaviour performance.^37^ ‘ *Opportunity costs*’ indicates the value of the next best alternative foregone because of the intervention. Lastly, ‘ *experienced intervention burden*’ stands for the amount of effort that is required to participate in the intervention. Acceptability usually looks at the introduction of a new device/technology^15,38,39^ or innovative ways to improve service delivery.^40-43^ Acceptability assessment can take place ‘ *before*’ the implementation of the healthcare intervention; during the intervention period, ‘ *concurrent assessment*,’ or after the implementation, ‘ *post-intervention*.’^22,23^ The present study has been done after implementation of Jazia PVS as a ‘ *post-intervention* assessment.’

**Table:** Theoretical Framework of Acceptability (TFA)

**TFA**	**Definition **
Perceived effectiveness	Experienced effectiveness: the extent to which the intervention is seen to have achieved its intended purpose
Affective attitude	Experienced Affective Attitude: How an individual feels about the intervention, after taking part
Intervention coherence	The extent to which the participant understands the intervention, how it addresses their condition, and how it works
Ethicality	The extent to which the intervention has a good fit with an individual’s value system
Self-efficacy	The participants’ confidence that they can perform the behaviour(s) required to participate in the intervention
Opportunity costs and Experienced burden	Experienced opportunity cost: the extent to which benefits, profits or values were forfeited to engage in the intervention Experienced burden: the amount of effort that was required to participate in the intervention (eg, participation requires too much time or expense, or too much cognitive effort)

Source: Sekhon et al.^23^

###  Study Area 

**Figure 1:**
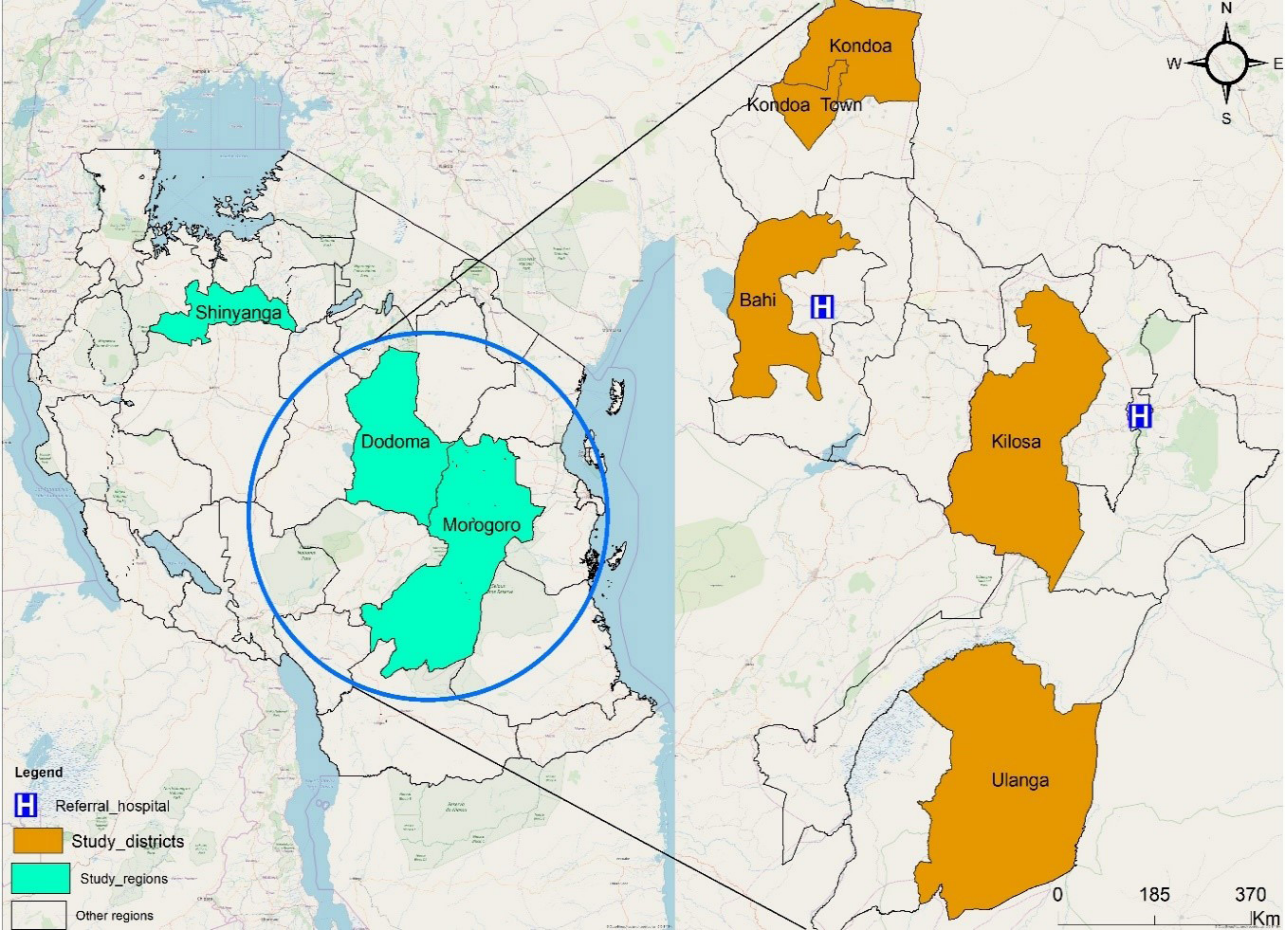
Study Regions and Districts.

We purposively selected 4 districts (Bahi and Kondoa districts in Dodoma region, and Kilosa and Ulanga districts in Morogoro region) out of thirteen districts where the Jazia PVS pilot has been implemented since 2014 and 2016, respectively. Districts selection was based on the high/low use of the prime vendor and distance from the regional Jazia PVS coordinating office. We included 2 districts with high use of the prime vendor and 2 districts with low use of the prime vendor. Monitoring and evaluation reports showed that over 18 months of Jazia PVS implementation, Kondoa and Kilosa districts had high use of the prime vendor while Bahi and Ulanga had low use of the prime vendor. In each region, we selected a district far away [more than 100 km] from the regional Jazia PVS coordinating office, while the other was located proximal to the regional office [less than 100 km] (Figure 1). Out of the 4 districts, 27 facilities (3 district hospitals, 8 health centres and 16 dispensaries) were selected in collaboration with district and regional managers, based on documented prime vendor use of facilities and proximity to the district headquarters.

###  Study Design 

Between July and September 2018, we conducted 30 qualitative in-depth interviews (IDIs), 7 group discussions (GDs) and 14 focus group discussions (FGDs). The FGDs included between 5 and 8 participants, while GDs had 2 to 4 participants. The length of the FGDs session was one and a half to 2 hours, while that of GDs and IDIs lasted 45 to 60 minutes. IDIs participants were purposively selected to represent the range of different actors involved in Jazia PVS implementation from the regional level, including the President’s Office for Regional Administration and Local Government (PO-RALG) (IDI 2); members of regional health management teams (IDI 2); regional level HPSS project managers (IDI 3 and GD 2); Jazia PVS Regional Coordination Office (IDI 2); CHMT members (IDI 6 and GD 1); Council Health Service Board (IDI 1); district accountants, auditors and procurement managers (IDI 5); healthcare facility in-charges (IDI 7 and GD 4); prime vendor representative (1); Jazia PVS consultant (1) and Health Facility Governing Committee (HFGC, FGD 14) (see Supplementary file 1, Table S1 and S2). In IDIs and GDs, we assessed the participants’ in-depth understanding of Jazia PVS and its acceptability after participating in the intervention. In the FGDs we explored participants’ general opinion of the acceptability of Jazia PVS as compared to the previous system of procuring complementary pharmaceutical supplies from different private suppliers.

###  Data Collection

We collected information on the acceptability of Jazia PVS based on the acceptability dimensions (Table). The IDI, GD and FGD guides were developed in English and later translated into the local language, Swahili. The Swahili version of the guides was piloted in 2 healthcare facilities (a dispensary and a health centre). The pilot results were used to refine the final data collection guides before use in the actual data collection activities. The first author and 2 research assistants, experienced in qualitative data collection activities, conducted the interviews. The first author took responsibility for organising the interview, welcomed the participants, moderated the discussion, and probed for additional information, while one of the research assistants asked questions and the other took field notes. We tape-recorded all the conversations during the fieldwork.

###  Data Management and Analysis

**Figure 2:**
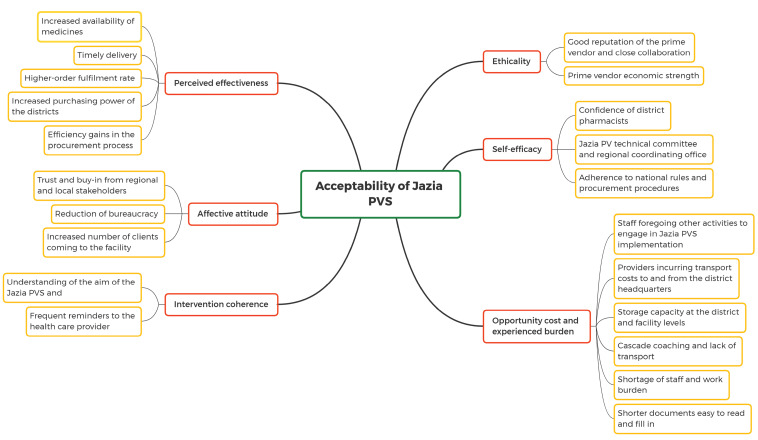
Acceptability of Jazia PVS. Abbreviation: Jazia PVS, Jazia prime vendor system.

FGDs, GDs, and IDIs were transcribed verbatim within 48 hours of the time they were conducted to allow follow-ups on emerging issues and points of clarification during the subsequent interviews. The transcribed data were reviewed and crosschecked for quality before they were imported into NVivo 12.0 (QSR International Pty Ltd). All the transcripts were analysed in their original language. Open coding was used in labelling, defining as well as developing categories based on dimensions of the participants’ descriptions. Subsequently, inductive as well as deductive approaches were used to group the codes into themes reflecting the acceptability dimensions.^22,23,44^ The first author and a senior social scientist performed intercoder reliability to improve the codes. The whole analysis focused on emerging themes, patterns, similarities, and differences. Framework analysis was used to summarise the results^45,46^ (Figure 2). The selected verbatim key quotations were translated into English and incorporated in the manuscript. We base the analysis on so-called data source triangulation which allows for data validation and comprehensive understanding of the Jazia PVS.^47-49^ In addition, it allows for exploration of the similarities and differences in terms of participants’ prior-expectations of Jazia PVS, or experienced and emotional reactions during and after implementation of the intervention.^47^

## Results

###  Perceived Effectiveness

 The ‘perceived effectiveness’ corresponds to the extent to which a given intervention has achieved its envisioned purpose. Sub-themes include increased availability of medicines, timely delivery and higher order fulfilment rate, increased purchasing power of the districts, and efficiency gains in the procurement process.

###  Increased Availability of Medicines, Timely Delivery, and Higher-Order Fulfilment Rate

 The study participants felt that the availability of essential medicines improved after implementation of Jazia PVS in the pilot regions. During the discussions with the regional and district managers, it was reported that, at the time of data collection (mid-2018), the average availability of essential medicines for some facilities was 90% to a 100%. One of the district officials elaborated:

“… *before the Jazia PVS, availability of medicines at the health facilities was so low. The average availability was between sixty to seventy percent, and could even be below sixty percent. After the introduction of Jazia PVS, the average availability of essential medicines increased up to eighty-ninety percent within the district*…” (ID I7, CHMT, Dodoma).

 Regional implementers highlighted that among the reasons for the establishment of Jazia PVS was the failure of the MSD to supply all the facility needs in each quarter. District managers felt that the ability of the prime vendor to supply medicines that matched the facility’s needs, contributed to the acceptability of Jazia PVS. When a regional implementer was elaborating on this, she said:

“… *Jazia PVS has an order fulfilment rate of a ninety-nine percent to a hundred, in case if a facility request ten tins, the vendor delivers them as required. Though, at times out of three hundred medicines ordered, two may be missing, but the vendor supplies them within two weeks…*” (IDI 2, regional implementer, Dodoma).

 In a follow-up discussion at the facility level, the HFGC members were of the opinion that Jazia PVS was effective in ensuring timely delivery of the consignments. When explaining this, participants made a comparison with the previous system, noting that they used to experience delays of sometimes more than 2 months in receiving medicines at the facility level. They further noted that the Jazia PVS has resulted in timely delivery of the consignment at the facility together with a reduction in the lead time. In one of the FGDs with HFGC, the following was reported:

“… *Jazia PVS has helped us; we get medicines on time; in the past, there were some delays. At the moment, whenever you press an order for medicines, they are delivered on time, different from the past where one had to wait for some time…*” (FGD16, HFGC, Morogoro).

####  The Purchasing Power of the Districts and Price of Medicines 

 District managers reported that the process of pooling facility orders at the district level to purchase from the prime vendor was seen as an efficient way to purchase medicines at lower than private market prices. They also felt that prices are fair, and at times they could negotiate commodity prices, and request the inclusion of additional items which are not listed in the contract. In discussion with the Jazia PVS implementers, it was further reported that, prior to the Jazia PVS, facilities purchased medicines from different suppliers at various price levels in each procurement cycle. District managers felt that increased transparency, pooling of all facility orders, and purchasing in bulk from the prime vendor with uniform medicine prices across facilities contributed to the acceptability of the Jazia PVS. This was detailed in the discussions with the district managers as follows:

“…. *I can say the system has increased the purchasing power of the districts, the prices are fixed all over for the customers. The notion of a certain health facility to purchase a tin of Paracetamol for eighty thousand, while another facility purchases the same tin for fifty thousand is no more, and we are all treated the same, this has increased the purchasing power of the districts*…” (IDI 10, CHMT, Kondoa, Dodoma).

 At the district level, managers confirmed that within the Jazia PVS, prices for the commodities supplied to the facilities are fixed, based on a contractual agreement with the vendor. In a follow-up discussion with the in-charges, they reported that facilities were paying high prices for the commodities before the implementation of the Jazia PVS. They further highlighted that in the previous system, suppliers used to inflate the price of the commodities each quarter to their benefit. When discussing cost savings with the in-charge, this was said:

“…. *prices for medicines are within the contract, different from the previous system. The list of all the items with which the vendor was awarded the contract was brought to us, and we use the prices through the contract duration which is two years*…” (IDI 13, Facility in-charge, Dodoma).

####  Efficiency Gains in the Procurement Process 

 Jazia PVS regional implementers described that, before its implementation, the staff at the districts spent an enormous amount of time in the process of purchasing complementary supplies. They further explained that, in each quarter, district officials travelled to Dar es Salaam or other regions looking for quotes from different suppliers which could take up to 3 weeks. The regional implementers highlighted that Jazia PVS rendered travelling in search of quarterly quotations and meetings unnecessary. This was reported in discussions with the regional implementers. For instance, one of them said:

“… *in 2011-2012 we had a shortage of medicines, the pharmacist travelled to Dar es Salaam for like three weeks. The pharmacist just came back with quotations and had been paid per-diem. Documents would then enter into the procurement cycle, and the facility could get medicines after a month. Jazia PVS has helped us a lot, and as implementers, we have seen its success*...” (IDI 2, Regional Implementer, Dodoma).

 In one of the districts, managers felt that the prime vendor is cost-conscious when delivering consignments to the respective districts. Besides, the manager noted that the vendor tended to combine orders for districts which are close to one another to control the operational costs. In explaining the vendor’s control of operational costs, this was said:

“ *At the beginning, districts were not ordering medicines together. Each district orders at its own time. To reduce operational costs, the vendor could tell us… ‘I will bring your consignment after few days’ or ‘I will bring it after three to four days because I want to combine and deliver orders for district A, district B and C which are close to each other’…”* (IDI 14, CHMT, Dodoma).

###  Affective Attitude 

 ‘Affective attitude’ refers to an individual’s feeling after taking part in the intervention. Sub-themes include trust and buy-in from regional and local stakeholders, reduction of bureaucracy, and increased number of clients coming to the facility.

####  Trust and Buy-in From Regional and Local Stakeholders 

 In discussions with representatives from PORALG, they felt that Jazia PVS has improved medicine availability at the facilities, and that is why the government decided to scale it up to other regions of Tanzania. They further highlighted that the decision was informed by its perceived effectiveness, including improved medicines availability at the facilities, reduction in the lead time [time from the facility ordering to the time of receipt of consignment at the facility], reduction in opportunities for fraud, and improved transparency in the whole system of procuring complementary supplies for the facilities. The buy-in from the government to institutionalise the Jazia PVS idea was also reported by district managers. They mentioned that the government recognized the achievement made by the system which is why it decided to roll it out.

“…. *based on the achievement resulting from the Jazia PVS, the government felt that Jazia PVS is a good system and should be adopted in all the regions of Tanzania*…” (IDI 19, CHMT, Morogoro).

 Trust in the Jazia PVS was also related to its private status and its clear rules on price adjustments. Some district managers felt that the prime vendor is profit-oriented and therefore able to obtain lower prices. District managers were of the opinion that the prime vendor was able to purchase directly from the manufacturers, offering the opportunity to bargain prices which, in turn, allowed the prime vendor to sell medical commodities to facilities at a lower price while still making a profit. In addition, it was reported that price adjustments were only allowed to offset inflation, based on the evaluation of a technical committee. While elaborating on price adjustment, one of the district officials explained:

*“...In the year 2015, there was inflation; the vendor requested for price adjustment. The contract allows for a price adjustment of the items whenever there is inflation. The vendor wrote a letter to request for a price adjustment on some of the items within the contract”* (IDI 14, CHMT, Dodoma).

####  Felt Reduction in the Bureaucratic Process During the Procurement 

 Regional and local stakeholders felt positive that the implementation of the Jazia PVS had resulted in a substantial reduction of bureaucratic procedures in the procurement process of complementary pharmaceutical supplies in the districts and at facilities. They reported that before the implementation of Jazia PVS, in each quarter it required more than fifteen signatures for authorising documents, and so the approval of documents took a long time. To clarify this point, a district manager explained:

“…. *Jazia PVS has reduced bureaucracy because in the past, once the facility received out of stock items from MSD, the facility in-charge had to write a letter to the district executive director, which took some time. Within the Jazia PVS, once you have documentation from MSD on missing items, the in-charge prepares the order and shares with the prime vendor through the district pharmacist*…” (IDI 25, CHMT, Morogoro).

####  Increased Number of Clients Coming to the Facility 

 In most of the facilities visited, it was reported that before the implementation of Jazia PVS, the number of clients accessing care in the facilities had been low. Healthcare providers felt that the prime vendor has led to an increased number of clients coming to their healthcare facility due to improved availability of essential medicines. Providers noted that clients are also coming from distant villages seeking care from their facilities because of improvement in service delivery and because clients could get all essential medicines prescribed to them. HFGC were of the opinion that there had been an improvement in the availability of essential medicines, causing more clients to seek care. In discussion with the HFGC members, this issue was mentioned as follows:

“…. *Many clients are coming to seek healthcare services because the facility has enough medicine and staff’s language is good compared to the past*.…” (FGD5, HFGC, Dodoma).

###  Interventions Coherence 

 ‘Intervention coherence’ implies how individual participants comprehend the intervention and how it works. Two sub-themes emerged: understanding of the aim of the Jazia PVS and frequent reminders to the healthcare provider.

####  Understanding of the Aim of the Jazia PVS 

 When asked about Jazia PVS coherence, regional and district managers showed a clear understanding of Jazia PVS’s purpose in strengthening the health system. They described that Jazia PVS was not a parallel system, rather it was meant to complement whatever was missing (out-of-stock) from MSD. Regional implementers highlighted that the MSD could not fulfil all facility needs each quarter; therefore, they felt that the Jazia PVS would complement the remaining facility needs. In discussing this issue, a regional manager said:

*“The purpose of the Jazia PVS was not to replace the MSD, as it could supply over half of the facility needs. So the prime vendor was designed to complement MSD, that’s why it was named ‘ Jazia,’ meaning that it fills the gap of out of stock from MSD*…” (GD1, Regional implementer, Dodoma).

 Conversely, the majority of the health facility in-charges were able to demonstrate how Jazia PVS operates, rather than stating its purpose. In describing the process of procurement of the commodities from the vendor, facility in-charges reported that the foremost task was to review the list of missing items (out of stock) from the MSD and funds available in the facility bank account. Facility in-charges explained that they first quantify the commodities to be purchased from the prime vendor, then they discuss with the HFGC for approval and share this with the district pharmacist. This process is explained by the facility in-charge, who said:

“… *once we have received a list of missing items from MSD, we discuss with the members of the health facility governing the committee. Then we prepare meeting minutes based on the facility needs, and submit to the district pharmacist, who reviews and shares directly with the prime vendor…”* (IDI 26, Facility in-charge, Morogoro).

####  Frequent Reminders to the Healthcare Provider 

 District managers reported that, at the beginning of the Jazia PVS, they spent a substantial amount of the time reminding providers about the quantification process and the need to prepare a list of their facility’s need just after receiving the notification of out-of-stocks items from the MSD. They went further and described that not all the facilities submitted their facility’s need list on time, so they had to remind the facility staff of the deadline to submit the needs because they had a lot of work to do at the facilities, including outreach services. It was noted that they have been using the ‘WhatsApp’ application platform in which they have formed a group for all the facility in-charges with smartphones to remind them on matters related to Jazia PVS. In clarifying about the frequent reminders and communication process with the facility in-charges, the manager had this to say:

“… *majority were delaying in quantifying facility needs at the beginning after receiving out of stocks from MSD, so we kept on pursuing them and reminding them that once you receive missing items, you should immediately start the process of ordering from the prime vendor*…” (GD2, CHMT, Dodoma).

###  Ethicality

 ‘Intervention ethicality’ indicates the extent to which the intervention has a good fit with an individual’s value system. Sub-themes include the good reputation of, and close collaboration with, the prime vendor, and the prime vendor’s economic strength.

####  The Good Reputation of the Prime Vendor and Collaboration

 Regional and district level stakeholders mentioned the good reputation of the prime vendor as a factor that promoted acceptability of the new system. They also felt that as a private supplier, the vendor supplies products of high quality, and the commodities are handled properly to protect the health of the consumers. District officials further highlighted the vendor’s commitment to observe the contractual agreement with the region. In discussing the good reputation of the prime vendor, a district official said:

“ *Professionally, I am a procurement person, and I know challenges associated with the procurement process. The prime vendor is committed and has the ability to meet the deadline, and has uniform prices”* (IDI 16, District official, Morogoro).

 District managers commended the Jazia PVS for its close cooperation with district officials, to the extent that, at times, they received support with regard to medical commodities from the vendor. Even the prime vendor’s marketing strategies were perceived as collaborative in nature by the district managers. For instance, they highlighted that on 2 occasions, the vendor had supported the district by sponsoring some medicines and medical equipment during community outreach activities and public gatherings. When explaining how close collaboration with the vendor works, the district manager said:

“ *Sometimes they provide free of charge some of the equipment, for example, haemoglobin test strips, with the expectation that districts and healthcare facilities will then purchase reagents because these are distributed by the vendor. At times, a vendor decides that, instead of selling the strip, he provides it for free*” (IDI 10, CHMT, Dodoma).

####  Prime Vendor Economic Strength 

 In one of the districts, the officials thought that the system is effective because the vendor has sufficient capital to run the business, compared to the previous system where suppliers had little capital. The district manager noted that before the implementation of Jazia PVS, it had been challenging to find a supplier who could supply complementary medicines for the whole district at once. In discussing the ability of the prime vendor, this was said:

*“…the district is so large, and previous suppliers had low capital, so it was difficult to get one supplier who could supply for all districts. The prime vendor has enough capital, that is the advantage; hence, the district can purchase as a whole, and receive most of the items as ordered”* (IDI 28, CHMT, Morogoro).

###  Self-efficacy 

 ‘Self-efficacy’ refers to the participants’ self-confidence that they can perform the behaviour(s) required to participate in the intervention. Three sub-themes emerged, including the confidence of district pharmacists, of the Jazia PVS technical committee and regional coordinating office, and adherence to national rules and procurement procedures.

####  View of District Pharmacists

 District pharmacists believed they could perform all procedures needed to ensure that the Jazia PVS attains the intended objectives. Pharmacists in the study districts reported communicating with the facility in-charges to make sure that they prepare their orders correctly and on time, and to ensure proper documentation of commodity usage at the facility. They confirmed that timely consolidation of requests from the facilities and prompt communication with the vendor is necessary for the system to function well. Pharmacists were responsible for the inspection of the consignment at the district level, before informing facility in-charges to collect their consignment. When a pharmacist was asked about his role, he reported that:

“… *as a district pharmacist my role is to ensure the system works properly, first making sure that facilities including district hospital, health centres and dispensaries prepare their orders on time. I communicate with a healthcare facility to get feedback on the quality of commodities procured from the vendor*…” (IDI 25, CHMT, Morogoro).

####  Jazia PVS Technical Committee and Regional Coordinating Office 

 Regional implementers at the regional Jazia PVS coordinating office expressed confidence in their ability to organise, monitor, and advise to ensure that the system performs as expected. Also, they reported that the regional coordinating office conducts quarterly monitoring and evaluates the vendor’s performance; reports are shared with the Jazia regional technical committee, the board, and regional administrative secretary (contract holder) for review during semi-annual meetings. A regional implementer stated:

“… *the first role of regional coordination office is to inform contract holder day to-day operations of the system if the system is performing well or not. It is also responsible for solving any conflicts emerging between the vendor and the districts, also responsible for compiling data coming from the districts…is also involved in monitoring and evaluation…prepare the report and submit to the technical committee*…” (IDI 1, Regional implementer, Dodoma).

####  Adherence to National Rules and Procurement Procedures 

 Regional and district managers felt that the prime vendor was effective as it followed all the procurement rules and regulations within the country. They further highlighted that during the selection of the vendor, stipulated procurement guidelines were followed, whereby all the potential retail and wholesale suppliers were invited and participated in the tendering process. Prime vendor technical committee members highlighted that they managed to review documents from the bidders and make sure that the selected prime vendor was licenced and registered with Tanzania’s pharmacy council, paid all the necessary charges (including taxes) to the government, and that the medicines supplied were registered by the Tanzania Food and Drug Authority. When discussing procurement laws and regulations with the district procurement managers, one of them said:

“… *the law directs us that to award any tenderer/vendor any work in which public funds are used, they must adhere to procurement rules and regulations, that is the work that we have been doing: ensuring they follow the rules and regulations*…” (IDI 3, Regional implementer, Dodoma).

###  Opportunity Costs and Experienced Burden

 ‘Opportunity costs’ connotes the benefits, profits, or values that were forfeited to engage in the intervention. ‘Experienced intervention burden’ refers to the amount of effort required to participate in the intervention. The section has the following sub-themes: staff foregoing other activities to engage in Jazia PVS implementation; providers incurring transport costs to and from the district headquarters; storage capacity at the district and facility levels; cascade coaching and lack of transport; shortage of staff and work burden, and shorter documents easy to read and fill in.

####  Staff Foregoing Other Activities to Engage in Jazia PVS Implementation 

 As described earlier, efficiency gains were perceived as relevant for the acceptability of Jazia PVS. However, the burden of Jazia PVS implementation on health personnel was mentioned as a challenge. In one of the regions, the manager reported that some of the staff who have been participating in the technical meetings have multiple tasks, and at times they have to forego other activities to participate in the implementation of Jazia PVS activities. Besides, the manager highlighted that having staff who are specifically employed for the Jazia PVS could improve the performance of the system, even beyond what people expect. In discussing the implications for other activities, the manager said:

“… *I am a specialised doctor, and at times I am supposed to go and provide treatment to the clients while, at the same time, I should participate in Jazia PVS monitoring and evaluation activities. If there were staff who were employed specifically for Jazia PVS activities, perhaps the efficiency of the system could go beyond what we expect…*” (IDI 2, Regional implementer, Dodoma).

####  Providers Incurring Transport Costs to and From the District Headquarter 

 In a few health facilities, in-charges reported incurring transport costs when submitting their order to the district pharmacists as well as when collecting the facility consignment. They reported spending a whole day for travelling to the district headquarters and submitting an order as well as for picking up the facility consignment. A few facility in-charges reported that incurring costs are not refunded when using a motorcycle to pick up the consignment at the district headquarters. A facility in-charge stated:

“… *we are supposed to receive medicines on time, in case of delays it becomes a problem, there comes a time as in-charge you are supposed to use your common sense if you have a motorcycle you go and collect facility consignment*…” (IDI 26, Facility in-charge, Morogoro).

####  Storage Capacity at the District and Facility Levels 

 In one of the districts, the manager reported that with the bulk purchases from the vendor, they faced a challenge with storage facility. The manager noted that the vendor delivers the whole consignment to the district level, and one of the problems is low storage capacity, because all the medicines should be stored appropriately before distribution to the respective health facilities. One of the district officials explained:

“… *one of the challenges is that our building is small and is not enough for the storage of the medicines. They are supposed to be kept in a standard form, and there is not enough storage space in our building…*” (IDI 25, CHMT, Morogoro).

 In a follow-up discussion at the facility level, in 2 of the facilities surveyed staff reported experiencing challenges with the storage capacity, in terms of room size as well as the nature of the shelves. The in-charge described that the facility had been built many years ago and that the size of the store and shelves were small compared to the volume of the consignments. In-charges highlighted that they have been receiving more drugs at the moment compared to the time before the implementation of the Jazia PVS. To clarify this point at the facility level, the in-charge said:

“… *at this time we have been receiving many drugs. However, the facility was constructed during the colonial period, and the room for the storage of medicines is small, so storage capacity has become a problem*…” (IDI 26, Facility in-charge, Morogoro).

####  Cascade Coaching and Lack of Transport 

 In discussions with district managers, it was reported that, during supportive supervision, staff who had experience and knowledge of commodity management were identified from health centres and dispensaries. They were trained in integrated logistic supply and offered the responsibility of peer coaching (mutual learning partnership among healthcare workers within the Jazia PVS pilot region to help identify best practices and creativity for improving healthcare service delivery). District managers highlighted that coaches were assigned at least 3 dispensaries for peer coaching on pharmaceutical supply chains to ensure that staff at the facility properly prepared a list of healthcare commodity needs and saved appropriate records. Besides, the manager highlighted that the coaches were facing a challenge as some dispensaries were located far away and that there were no funds apportioned to undertake coaching. In discussing cascade coaching and the burden that coaches from the health centre were facing, the following was said:

“… *at the beginning we had staff from the health centre to coach staff at the dispensaries, it became difficult, for a person to serve about ten facilities which are in distant places. So, we looked at the performance of other facilities and re-distributed into three to four facilities. This helped and reduced the work burden*…” (IDI 14, CHMT, Dodoma).

####  Shortage of Staff and Work Burden 

 In 2 of the study districts, it was reported that there was a shortage of staff. District managers highlighted that the existing staff are assigned a lot of work which they have to complete and, at the same time, perform Jazia PVS activities. The district manager reported that they are responsible for the management of hospital medicine supplies. Additionally, receiving and reviewing most of the health facility reports was challenging for just one person to oversee the whole process. In explaining the shortage of workforce, and in view of other assigned duties such as reviewing store ledgers, voucher books, and bank balance, the manager had this to say:

“… *there is a shortage of staff at the district, and at times I am forced to work till five p.m. in the evening and may extend up to eight p.m. At the hospital, I am responsible in overseeing medicines for the hospital and at the same time receiving and reviewing reports from the facilities, so it becomes a challenge for someone to perform all the assigned tasks on time*…” (IDI 25, CHMT, Morogoro).

####  Shorter Documents Easy to Read and Fill in

 District managers felt that the implementation of the Jazia PVS came with additional paperwork at the facility level during quantification and inspection of the commodities. However, they maintained that the forms were not complicated and easy to fill in. They reported that facility in-charges were using the forms, which were easy to read and fill in during quantification and inspection of the consignment. The district official explained this process:

“… *to a greater extent, procedures at the facilities have been simplified so that staff at the facilities may follow easily. During the inspection of the consignment, there is a form that they fill in after receiving medicines which has simplified the process. Also, when quantifying the medicines to be procured from the vendor, there is a particular form facility in-charges have to fill in, it’s easy to read and fill in*…” (IDI 14, CHMT, Dodoma).

## Discussion

 This study analysed the acceptability of Jazia PVS in Tanzania among stakeholders involved in its implementation. Among the factors named most frequently as contributing to the acceptability of the Jazia PVS is the perceived effectiveness of the system. Participants felt Jazia PVS has been effective in increasing the availability of essential medicines at the facilities, in timely delivery of the consignment together with higher order fulfilment rates. Intervention coherence, a reduction in experienced opportunity cost, and low intervention burden were also important in explaining the acceptability of the Jazia PVS. Other factors included affective attitude and self-efficacy.

The study findings on the perceived effectiveness reflect the extent to which Jazia PVS has achieved the intended purpose of improving the availability of essential medicines in public healthcare facilities. The results are consistent with other studies which have found perceived effectiveness of an intervention in solving a problem or making improvements to be among the factors explaining acceptability of the intervention.^15,27,43^ In Malawi, perceived effectiveness of short message service, web-based reporting and a resupply system resulted in reducing stock-outs of healthcare commodities, thus influencing the acceptability of the intervention.^15^ In South Africa a prime vendor system resulted in improved availability of medicines at the facilities, increased reliability, and cheaper distribution; however, acceptability of the system was not assessed.^50^

Similarly, other studies conducted in different settings found that pooling of healthcare facility orders increased the purchasing power of health providers, and district and regional managers were able to negotiate prices for the commodities due to economies of scale.^51-54^ Similarly, in Thailand, a collective provincial bargaining system across district hospitals and health centres resulted in a twelve to 20% reduction in drug prices.^54^ In Cameroon, pooled procurement of pharmaceutical supplies was considered the best option for increasing the volume and purchasing power of drug supply organizations, hence lowering the price of the medicines.^53^ Proper management of pooled procurement increases pharmaceutical procurement efficiency and in most cases actually does reduce transaction costs.^52,55^

We found that experienced intervention burden and lower opportunity costs related to the previous system influenced the acceptability of the Jazia PVS. The findings of this study are similar to those of an innovative programme which was conducted in Senegal and Vietnam to integrate the medical product supply chain for all public-sector vaccines, drugs, and other health products.^28,56^ After integration, vaccines and other medical commodities were allocated from the national level to the regional level. Acceptability of the intervention was influenced by time-savings for healthcare staff and caretakers in administering vaccination; the reduction in workload for staff involved the supply chain and improved the availability of vaccination and health products.^15,28,56^ In Malawi, Shieshia et al noted that the reduction in effort, time, and money spent in travelling to the districts to follow up the facilities’ needs contributed to the acceptance of the m-Health technology (“medical and public health practice supported by mobile devices”) in strengthening the community health supply chain.^15^

Self-efficacy within the context of the intervention contributes to effective implementation.^28^ Pharmacists and Jazia PVS technical committee members expressed their ability to manage day-to-day Jazia PVS activities, ensuring that the vendor complies with the contractual agreement and other laws/regulations. In Tanzania, various institutions are in place to oversee the operations of prime vendors for safety purposes, including the pharmacy board and the Tanzania Food and Drug Authority.^57^ Close monitoring of the prime vendor remains crucial to the supply chain; there were cases of regulatory infringements which were linked to the failure to implement sanctions by respective institutions, staff malpractices, and concealment of regulatory violations.^57^

Findings on the affective attitude and ethicality of the intervention show that participants had a positive feeling towards Jazia PVS. Murphy and Gardner assessed pharmacists’ acceptability of a men’s mental health promotion programme using the framework of Sekhon et al and found that participants’ acceptability of the intervention was influenced by their positive feeling that the programme would improve men’s mental health.^40^ In this study, regional and district managers felt that the programme resulted in a substantial reduction in bureaucratic procedures in the procurement process. Similarly, in Thailand, acceptability of the collective provincial bargaining system was influenced by the active involvement of the district hospitals, by the reduction of bureaucratic steps in the procurement process and by the absence of conflicts of interest.^54^

To the best of our knowledge, this was the first study to apply a theoretical framework of acceptability to assess the implementation of a pharmaceutical supply chain PPP intervention in a low-income setting. Nevertheless, the study contends with a few minor limitations. Not all acceptability dimensions could easily be analysed, such as affective attitude and ethicality of intervention. There could be some overlap with regard to some dimensions; for example, there is no clear demarcation between the opportunity cost experienced during the intervention and the intervention burden conferred by the intervention. In this study, as we assessed acceptability using self-reported experience with the Jazia PVS, there could be some social desirability recall bias.^58^ However, this was minimised by the purposive selection of participants and data triangulation.^47,49^

The field would benefit from future studies that use objective measures and individual self-reported measures to assess acceptability and compare the findings. Similarly, the methodological assessment of acceptability depends on the nature of the intervention being implemented. Several methods have been used to assess acceptability, including objective measures of behaviour (such as all-cause discontinuation, dropout rates or withdrawal rates), individual self-reported measures (such as satisfaction measures, attitudinal measures, personal experiences with the intervention), and both objective measures and individual self-reported measures.^23^ There is no established convention concerning the time for an assessment of acceptability, and one could conduct a study pre-intervention, post-intervention, or during the intervention. Future studies targeting the pharmaceutical supply chain could choose different time points for the purpose of comparison. Ongoing monitoring activities would enable identifying problems with acceptability over time and would facilitate comparisons of acceptability between alternative or competing interventions.^22^ In terms of the quality of medicine, the Jazia PVS programme has measures in place to ensure that the selected prime vendors deliver healthcare commodities of high quality to facilities. However, this study did not capture and track changes in the quality of medicines supplied by the prime vendor. Furthermore, this study did not explore the perceived effectiveness on the part of staff and executive management of the MSD, the institution mandated with the supply and distribution of essential medicines in the public sector in Tanzania. Their opinion and experience of the effectiveness of the Jazia PVS would have enriched the findings of this study and strengthened policy recommendations.

## Conclusion

 The findings of this study indicate that the perceived effectiveness of the Jazia PVS was mentioned most frequently by the participants in explaining its acceptability. Jazia PVS was perceived to be effective in improving the availability of essential medicines at public healthcare facilities, a key determinant of access to healthcare. Districts purchasing directly from the prime vendor have the well-structured and institutionalized possibility to increase availability of essential medicines in peripheral areas in a low-income setting. However, when implementing this strategy, it is crucial to select a reputable and competent vendor that abides by contractual agreements. As in any PPP, these contractual obligations need to be followed by both parties. In addition, it is important to establish and maintain a routine monitoring system which will allow tracking the effects and results of an innovation such as Jazia PVS on the pharmaceutical supply. Furthermore, it is important to strengthen coordination between the entities responsible for managing the supply chain of healthcare commodities at different levels to ensure timely availability and proper use of health commodities at the facilities.

## Acknowledgements

 We would like to acknowledge the assistance of the HPSS project in Dodoma Tanzania. We would like to thank the regional and district managers together with the facility-in-charges within the sampled facilities for their support during the study. We would like to thank Dr. Marina Antillon for her critical review and proofreading the paper as well as Mr. Yeromin Mlacha for his support in generating the image for visualization of the study regions and districts. We also acknowledge the Swiss Programme for Research on Global Issues for Development (r4d); SDC; Swiss National Science Foundation (SNF) for funding the study.

## Ethical issues

 The Medical Research Coordinating Committee of the National Institute for Medical Research in Tanzania (NIMR/HQ/R.8a/Vol. IX/2720) together with the Ifakara Health Institute (IHI) Institutional Review Board (IHI/IRB/No. 21-2017) approved the study. We obtained written informed consent from all study participants. We assured confidentiality and anonymity in the process of collection, cleaning, analysis, and reporting of the study findings.

## Competing interests

 Authors declare that they have no competing interests.

## Authors’ contributions

 AK and FT conceptualized the study. AK participated in data analysis, interpretation of data, and drafted the manuscript; VSM substantially contributed to data acquisition. KWi, BO, and EM participated in interpretation of results. KWy and FT contributed to interpretation of results and in writing the manuscript. All authors have critically reviewed the manuscript and approved the final version.

## Funding

 This manuscript is an output from the project: Health systems governance for an inclusive and sustainable social health protection in Ghana and Tanzania funded by the Swiss Programme for Research on Global Issues for Development (r4d programme, phase one March 2016 – March 2019). The project involved a consortium of 5 partners: Swiss Tropical and Public Health Institute, ETH Zurich, University of Applied Sciences and Arts of Southern Switzerland (SUPSI), Ifakara Health Institute Tanzania, University of Ghana.

## Authors’ affiliations

^1^Ifakara Health Institute, Dar es Salaam, Tanzania. ^2^Swiss Tropical and Public Health Institute (Swiss TPH), Basel, Switzerland. ^3^University of Basel, Basel, Switzerland. ^4^School of Public Health and Social Sciences (SPHSS), The Muhimbili University of Health and Allied Sciences (MUHAS), Dar es Salaam, Tanzania. ^5^Health Promotion and System Strengthening (HPSS) Project, Dodoma, Tanzania.

## Supplementary files

Supplementary file 1 contains Tables S1-S2.
