# Supplementary material for: Acceptability of a Prime Vendor System in Public Healthcare Facilities in Tanzania
Source: Int J Health Policy Manag. 2020 Jun 14;10(10):625–37. doi: 10.34172/ijhpm.2020.90 (PMC9278535; doi:10.34172/ijhpm.2020.90)
Supplement: Supplementary file 1 — contains Tables S1-S2. [file ijhpm-10-625-s001.pdf]

Supplementary file 1

Table S1. Categories of respondents at the regional, district, and facility levels

Region	Dodoma Region						Morogoro Region					
Council	Kondoa DC			Bahi DC			Ulanga DC			Kilosa DC		
Category of Respondent	FGD	IDI	GD	FGD	IDI	GD	FGD	IDI	GD	FGD	IDI	GD
Council health management team (CHMT)		2			1	1		1			2	
District accountants/ auditors		1						1			1	
District procurement managers								1			1	
Health service providers		1			1	1		2	3		3	
Health facility governing committee (HFGC)	5			3			2			4		
Council health service board (CHSB)		1										
Total	5	5		3	2	2	2	5	3	4	7	

Table S2. Categories of respondents at the national, regional, and district levels

National and regional level key informants	**IDI**	**GD**
Health Promotion System Strengthening (HPSS)	3	2
President's Office for Regional Administration and Local Government –PO-RALG (Jazia coordinating office)	1	
President's Office for Regional Administration and Local Government –PO-RALG (Procurement department)	1	
Jazia PVS Regional Coordination Office	2	
RHMT members (Regional Pharmacists /Regional medical officer)	2	
Bahari Pharmacy representative	1	
Jazia PVS Consultant	1	
Total	11	2
